# A novel approach to CSF pressure measurement via lumbar puncture that shortens the measurement time with a high level of accuracy

**DOI:** 10.1186/s12868-023-00805-4

**Published:** 2023-06-18

**Authors:** Duygu Yücel, Yekta Ülgen

**Affiliations:** 1 Genome and Stem Cell Center (GENKOK), Erciyes University, Kayseri, Turkey; 2 Department of Medical Engineering, School of Engineering and Life Sciences, Acibadem University, Istanbul, Turkey

**Keywords:** Cerebrospinal fluid pressure, Diagnostic lumbar puncture, Neurological diseases, Spinal needle, Spinal manometer, Time constant

## Abstract

Intracranial pressure (ICP) is an important parameter in clinical management and diagnosis of several neurological diseases which is indirectly measured via lumbar puncture (LP). In routine measurements of cerebrospinal fluid pressure (*P*_*CSF*_) from lumbar region, a spinal needle and a spinal manometer are used. *P*_*CSF*_ measurement via LP with the use of a spinal manometer may not yield correct *P*_*CSF*_ results due to prolonged times required to obtain an accurate pressure value. Equilibrium pressure may be underestimated in circumstances where spinal manometry procedure is terminated prematurely, with the wrong assumption that equilibrium pressure is reached. Elevated *P*_*CSF*_ levels can lead to visual loss and brain damage when go undiagnosed. In this study, the spinal needle-spinal manometer combination was modelled with a first-order differential equation and a time constant (*τ*) was defined as the product of the resistance to flow of the needle with the bore area of the manometer divided by the dynamic viscosity of CSF, i.e. *τ*= RA/*ρ*_*CSF*_. Each needle/manometer combination had a unique constant as a predictor of the equilibrium pressure. The fluid pressure in the manometer rose in an exponential manner which was tested in a simulated environment using 22G spinal needles namely Braun-Spinocan, Pajunk-Sprotte and M.Schilling. Curve fitting of the manometer readings were obtained with regression coefficients of R^2^ ≥ 0.99 to determine measurement time constants. The residual differences between predicted and true values were less than 1.18 cmH_2_O. For a given needle/manometer combination, time required to reach equilibrium pressure was identical for all pressure levels. *P*_*CSF*_ measured at reduced times can easily be interpolated to their equilibrium level allowing clinicians to obtain *P*_*CSF*_ values with high accuracy within seconds. This method can be used as an indirect estimation of ICP in routine clinical practice.

**Supplementary Information:**

The online version contains supplementary material available at 10.1186/s12868-023-00805-4.

## Background

**Fig. 1:**
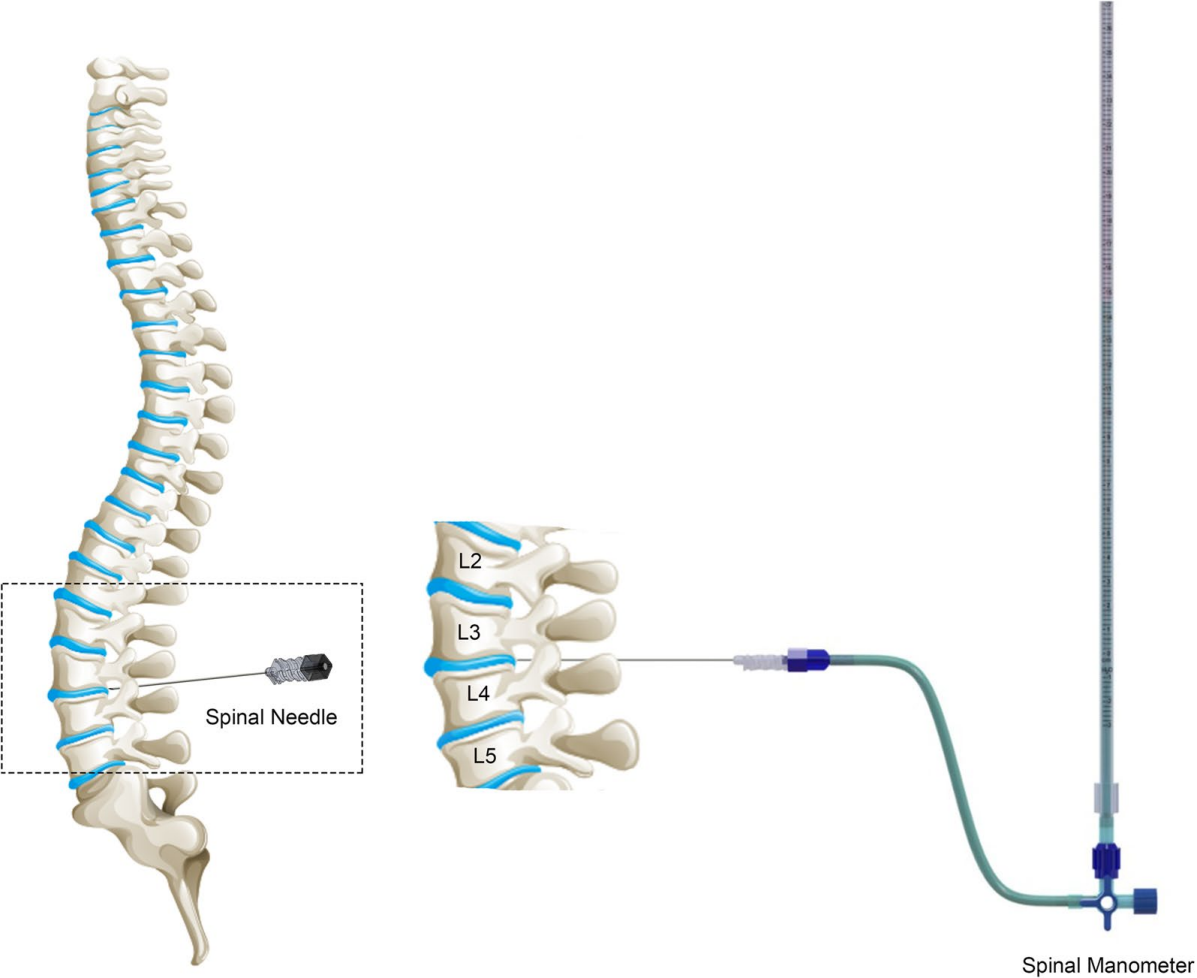
CSF pressure measurement via lumbar puncture is ilustrated. L2, L3, L4 and L5 indicate lumbar vertebra. In general, spinal needle is positioned at or below L3-L4 interspace to perform lumbar puncture. Spinal needle is connected with a spinal manometer to measure CSF pressure

Intracranial pressure (ICP) is an important parameter in clinical management and diagnosis of several neurological diseases including hydrocephalus, idiopathic intracranial hypertension (IIH) and demyelinating disorders [1]. The current clinical practice to measure ICP relies on two invasive techniques. First is a direct measurement by a pressure probe or micro transducer inserted into the intraventricular or parenchymal regions of the brain. This method involves opening the skull and requires neurosurgical expertise to penetrate through the brain. The second method is an indirect ICP measurement via lumbar puncture (LP) which is less invasive and has a lower infection risk compared with the ventricular or parenchymal ICP measurement methods yet presents with several disadvantages (Fig. 1) [2–4]. The cerebrospinal fluid (CSF) pressure (*P*_*CSF*_) from the lumbar region is classically measured with a spinal manometer [3, 5–8]. During diagnostic LP procedures where spinal manometry is used for *P*_*CSF*_ measurement, prolonged times are required to obtain an accurate pressure value. Two critical factors have impact on *P*_*CSF*_ measurements: the flow rate of CSF through the spinal needle and the needle response to transduce the 90% of *P*_*CSF*_ with high speed and accuracy. A quick and accurate assessment of *P*_*CSF*_ is essential for the health and the comfort of the patients. Equilibrium pressure may be underestimated in circumstances where spinal manometry procedure is terminated prematurely, with the wrong assumption that equilibrium pressure is reached. This assumption is due to very slow upward movement of the CSF in the manometer in the case where smaller-diameter spinal needles, that is ≥ 20G are used. Needles with larger inner diameter can transduce correct *P*_*CSF*_ value less than a minute yet the use of large-diameter needles have been reported as a risk factor for Post-dural Puncture Headache (PDPH) occurrence [9]. The time required for the fluid to reach to the top most level in the manometer is defined as “equilibration time” which is the time required to obtain a correct *P*_*CSF*_. Equilibration time of a given *P*_*CSF*_ is dependent on the inner diameter of the spinal needle and the bore diameter of the manometer that is the spinal needle-spinal manometer assembly which has not been investigated according to our knowledge.

Normal *P*_*CSF*_ values are 10–18 cm H_2_O in lateral position [10]. Refractory chronic headache patients may have mean peak CSF pressures as high as 39.8 cm H_2_O [11]. Elevated intracranial pressures corresponding to pressures larger than 25 cm H_2_O is a risk of mortality. Under various pathological conditions, the relationships between volumes of parenchyma, blood and CSF changes. An average adult produces approximately 500 ml of CSF daily which is replaced about four times a day indicating that approximately 20 ml of CSF is replenished every hour. CSF is mainly produced by the choroid plexus residing in the ventricles. CSF flow is determined by fine tuning of production and absorption of CSF. The measurement of flow rate *Q* of CSF through the spinal needle can accurately predict the intracranial pressure. Disruption of this delicate balance impairs the CSF flow rate resulting in diseases such as hydrocephalus. The pulsatility of the CSF flow have been demonstrated approximately four decades ago via magnetic resonance imaging (MRI) studies [12, 13]. The arterial input to the cranium is the main cause of the CSF pulsation [8, 14].

In this study, we hypothesized that spinal needle-spinal manometer assembly could be represented with a first-order differential equation. We have identified that each needle/manometer combination has a unique time constant. To our knowledge, this is the first time where spinal needle-spinal manometer assembly was defined with a first-order model revealing the fact that measurement times required for a given percentage of *P*_*CSF*_ are independent of the final equilibrium level and waiting for CSF to reach the equilibrium height is not necessary. This method has the potential to revolutionize routine diagnostic LP procedures allowing clinicians to obtain *P*_*CSF*_ values with high accuracy within seconds.

## Methods

### First-order model for PCSF measurements

**Fig. 2:**
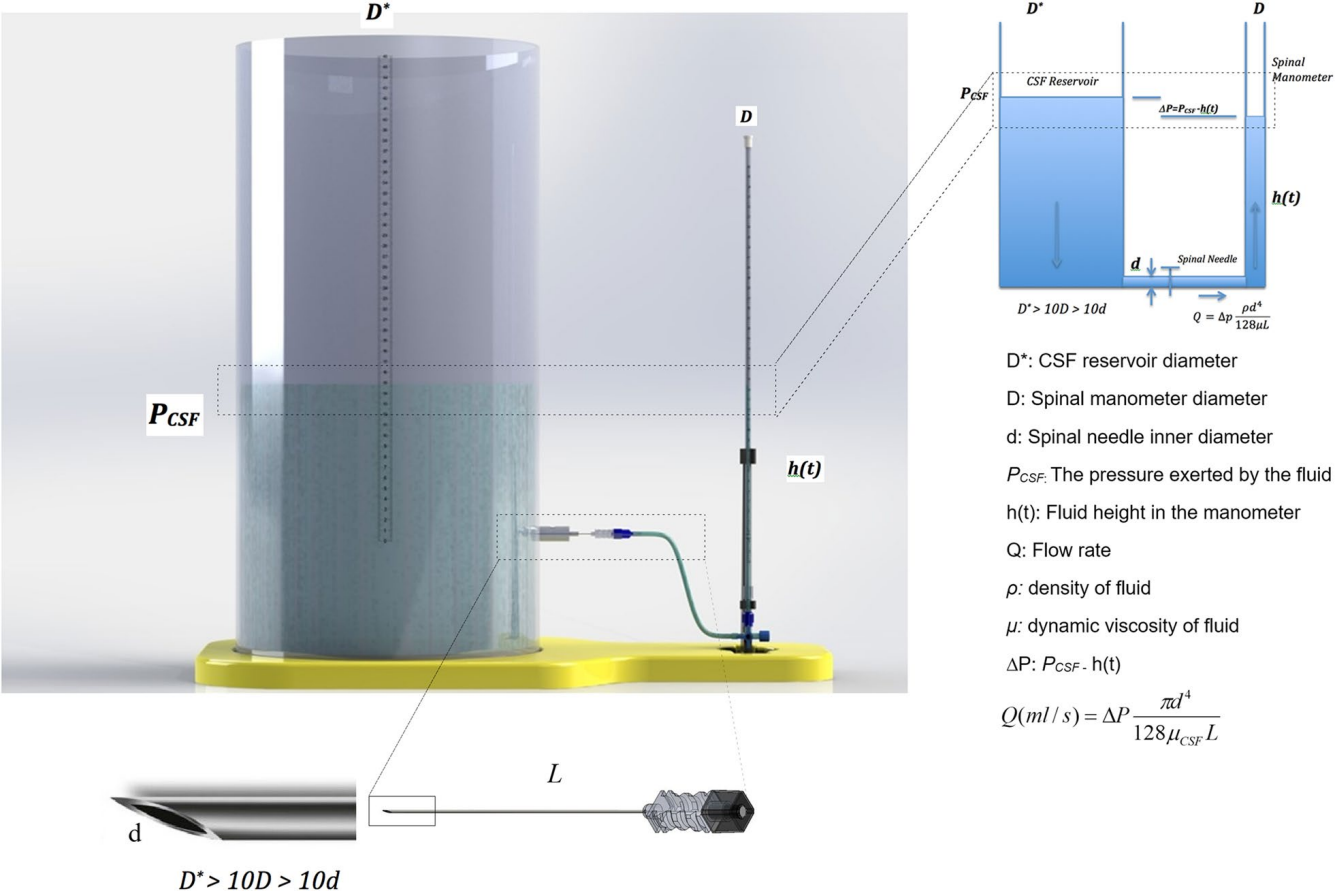
Illustration of the set-up used in simulated measurements. CSF reservoir, the tubing, the 3-way stopcock and the spinal needle—spinal manometer assembly are shown

The CSF measurement model we proposed, consisted of a large diameter CSF reservoir made to communicate with a spinal needle and a spinal manometer, as illustrated in Fig. 2. When referred to literature, the reservoir model in simulated CSF experiments was filled with artificial CSF, Ringer Lactate or 0.9% saline solutions of similar densities and viscosities with the CSF fluid. To create a reference fluid column of constant pressure P_CSF_, the reservoir tube diameter must be at least ten times the manometer diameter (D* ≥ 10D), in order to compensate for a loss of fluid volume in the reservoir. Reservoir with a diameter of at least ten times will result in a pressure drop of less than 1% in the manometer at equilibrium. [15–17].

The model behavior is governed by Pascal’s law of communicating vessels. As the spinal needle and the manometer connection is made, CSF runs up the manometer tubing, where the height of the fluid slowly but continuously fills the manometer until the pressure exerted by the fluid column *h(t)* in the manometer equals the cerebrospinal fluid pressure *P*_*CSF*,_ and then the flow stops. Capillary action of the manometer is omitted, since according to Jurin’s law of capillary action, a 3 mm bore tube will result in a negligibly small height increase of 5 mm only [18]. Clinical reference methods define the CSF equilibration time as the time required for the cerebrospinal fluid to rise to this constant height. The pressure difference between the cerebrospinal fluid pressure *P*_*CSF*_ and the pressure exerted by the CSF height *h(t)* in the manometer determines the CSF flow rate:

1 $$ Q = \frac{\Delta P}{R} = \frac{{\left( {{\text{P}}_{CSF} - h(t)\rho } \right)}}{R} $$

where R is the resistance to flow of the spinal needle and *ρ* the density of CSF. The flow rate can also be expressed as Q = dV(t)/dt, V(t) being the instantaneous CSF volume accumulated in the manometer:

2 $$ Q = \frac{dV}{{dt}} = \frac{d(Ah(t)}{{dt}} = \frac{Adh(t)}{{dt}} $$

Here, *A* is the inner cross-sectional area of the manometer tube. Combining Eqs. 1 and 2 together:

3 $$ A\frac{dh(t)}{{dt}} = \frac{{({\text{P}}_{CSF} - h(t)\rho )}}{R} $$

Rearranging,

4 $$ \frac{dh(t)}{{dt}} = \frac{\rho }{RA}h(t) = \frac{{{\text{P}}_{CSF} }}{RA} $$

This is a first-order differential equation and the standard form for the homogeneous solution is $\frac{dh(t)}{{dt}} = \frac{\rho }{RA}h(t) = 0$, with the time constant τ defined as:

5 $$ \tau \, = \,RA/\rho $$

This implies that, for each spinal needle-spinal manometer combination, there will be a unique characteristic time constant (*τ*) xpressed as the product of the resistance to flow and the manometer cross sectional area, assuming the density *ρ*_*CSF*_ is approximately 1 (= 1.0006 g/ml). The general solution to the system has then the form:

6 $$ h(t) = P_{CSF} (1 - e^{ - t/\tau } ) $$

This Equation implies that time taken for the manometer to reach a specified ratio *r* = *h(t)/P*_*CSF*_ of the equilibrium pressure height is independent of *P*_*CSF*_. The ratio r is 50%, 63%, 86%, 95% and 99% when measurement times are 0.693τ, τ, 2τ, 3τ and 5τ respectively.

### Resistance to flow (R)

The resistance to flow in plastic tubings is a well-known concept and never considered by the spinal needle manufacturers. Therefore, prior to pressure measurements, it is necessary to determine *R* by using one of the following method.

By opening one end of the needle to atmosphere via the 3-way stopcock in Fig. 2 and applying Poiseuille's Law of fluid flow dynamics, the laminar flow through the spinal needle (of length *L* and internal diameter *d*) was linearly related to the driving pressure *∆P*:

7 $$ Q(ml/s) = \Delta P\frac{{\pi d^{4} }}{{128\mu_{CSF} L}} $$

where *µ*_*CSF*_ is the dynamic viscosity of CSF and equals 1.002 mPa.s at 20 °C. The expression $\frac{{128\mu_{CSF} L}}{{\pi d^{4} }}$ represents the resistance to flow and can be determined by direct substitution of *µ*_*CSF*_* (cmH*_*2*_*O s),* the diameter* d* and the length *L* of the spinal needle.

The flow rate *Q,* under a given pressure *P*_*CSF*_, can be calculated by measuring the total number and total mass *m*_*CSF*_ of CSF drops (of constant rate) from the end of the spinal needle, over a fixed time period *∆t* [2, 19]:

8 $$ Q(ml/s) = \frac{{m_{CSF} (g)}}{{\rho_{CSF} (g/ml)\,\Delta t(s)}} $$

*ρ*_*CSF*_ is the density of CSF (from 1.005 g/ml to 1.006 g/ml) [4]. We employed Eq. 8 in calculating the resistances to flow:

9 $$ R{\text{(cm s/ml)}} = \frac{\Delta P}{Q} $$

### Laboratory measurements using a CSF model

To mimic the CSF pressure, a cylindrical reservoir of 5 cm diameter (D^*^) and 30 cm height, filled with Ringer lactate solution, was used. The condition D^*^ ≥ 10 D guaranteed a pressure drop of less than 1% at system equilibrium. The reservoir was made to directly communicate with the spinal needle and the spinal manometer using a tubing, as illustrated in Fig. 2. The spinal manometer (by Bıçakçılar, Turkey) had a bore diameter of 3.7 mm. According to Jurin’s law of capillary action, under ideal conditions, a 3 mm bore tube will result in a height of 5 mm only. Therefore, capillary action was neglected. The spinal needles were 88 mm long 22G Braun Spinocan with an external diameter of 0.70 mm; 90 mm long 22G Pajunk Sprotte with an external diameter of 0.72 mm and 90 mm long 22G M. Schilling with an external diameter of 0.70 mm. To determine the resistances to flow, drop weights were measured with a precision balance, and times between consecutive drops were recorded using a stopwatch, for finding the flow rate *Q* under the given pressure of 13 cmH_2_O (Eq. 8)_*.*_* R* was then directly determined from the formula *R* = *P/Q *(Eq. 9).

**Fig. 3:**
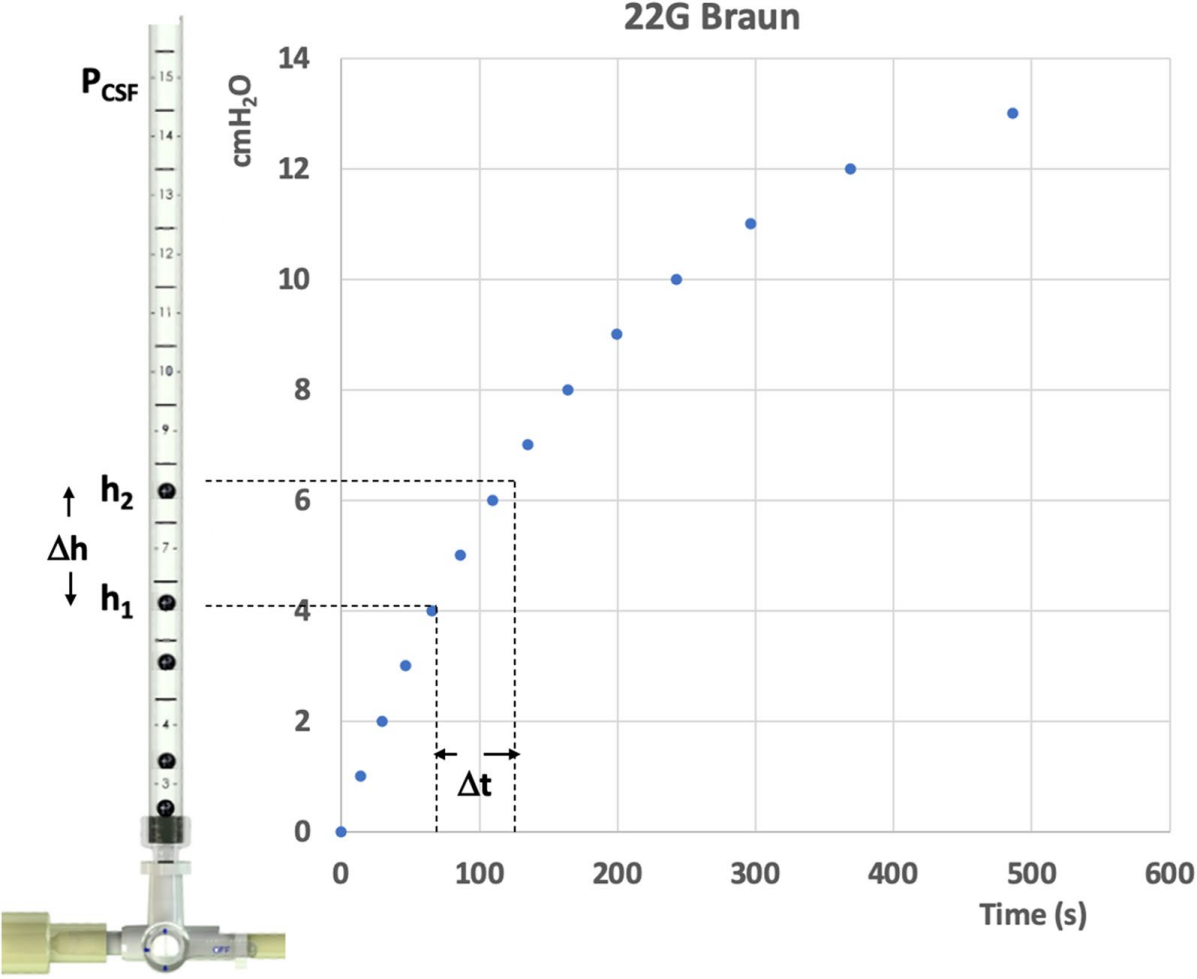
The CSF pressure rises in an exponential manner in the spinal manometer. Representative data for one of the needles is shown

Each needle-manometer combination was tested three times, using the simulated CSF set-up, by reading the incremental rise times (in steps of 1 cm) until a final height of 13 cm was reached in the manometer (Fig. 3).

The rise times were measured by first video filming the fluid rise in the manometer and then timing it with the lab chronometer. First-order differential equation of the form given by the Eq. 6 was verified by non-linear regression fitting for each needle. The theoretical equation was plotted by using the average times to pre-defined pressure heights, ranging from 0 cmH_2_O to 13 cmH_2_O.

### Frequency response

The model behaves as a low-pass filter with a 3 dB cut-off frequency at $1/2\pi \tau$. Taking typically *τ* = 50 s gives f_c_ = 0.0032 Hz. Hence, the measuring system will not respond to relatively faster CSF physiological rhythm changes caused by the respiratory (0.3 Hz) (20 breaths per minute) and cardiovascular (> 1 Hz) activities [20–22].

10 $$ \frac{H(jw)}{{P_{CSF} (jw)}} = \frac{1/\rho }{{(jw + \frac{\rho }{RA})}} $$

We previously compared pulsatile and non-pulsatile CSF models for CSF pressure measurement via LP where measurements were indifferent [2]. The cardiac variation was 1 to 2 mm CSF and the respiratory one only slightly greater, namely 2 to 5 mm CSF under normal breathing; 5 to 10 mm CSF with deep breathing, as observed at lumbar puncture. In a recent experiment, which used a pulsatile CSF model for driving the needle-manometer system, no perceptible change was observed in the pressure measurements [2, 22].

For monitoring the dynamic behavior of CSF, the spinal manometer was replaced with an electronic pressure transducer to detect negligibly small pressure changes (few mm) due to physiological effects [18, 23]. The ICP (normally 5 to 15 mm Hg) waveform however, unlikely the CSF pressure, is pulsatile and highly sensitive to respiratory and cardiac activities: respiratory waves varies between 2 to 10 mmHg and the cardiac component between 1 to 4 mm of Hg [24].

### Statistics

Simple nonlinear regression analysis (exponential, least squares fit) was used as a tool to quantify the relationship between pressure and time. GraphPad Prism 8.3.0. version was used in all analysis.

Curve fitting of the manometer readings were performed with regression coefficient R^2^ ≥ 0.99, when determining the measurement time constants.

## Results

Time constants for the 22G Pajunk Sprotte, the 22G Braun Spinocan and the 22G M. Schilling spinal needles combined with a manometer of 3,7 mm bore diameter were calculated. First, flow rate for each needle was determined using Eq. 8. *R* was then directly determined from the formula *R* = *P/Q* (Eq. 9). The measurements are shown in Table 1.

**Table 1:** Resistance to flow measurements

Type of needle	Measurement (M)	Total number of drops	Total weight of drops (mg)	Measurement time (s)	Flow rate (cmH2O)	R (cm.s/ml)	AVG + SD
22G Pajunk Sprotte	M1	10	573.60	25.5	0.0225	667.5	667.9 ± 0.91
	M2	10	574.20	25.6	0.0224	668.6	
	M3	10	590.80	26.3	0.0225	667.7	
22G Braun Spinocan	M1	10	549.60	51.8	0.0106	1415.0	1411.3 ± 3.49
	M2	10	539.50	50.7	0.0106	1410.8	
	M3	10	554.50	52.1	0.0107	1408.0	
22G M. Schilling	M1	9	480.80	32.6	0.0147	1017.1	1028.7 ± 11.3
	M2	9	508.60	34.9	0.0146	1029.3	
	M3	9	507.80	35.2	0.0144	1039.8	

Using the R from the Table 1, time constants for each needle—manometer combination was determined according to Eq. 5; i.e. *τ* = RA_._ The manometer used in this study had a bore diameter of 3,7 mm which was used to calculate area (A). The time constants for 22G Pajunk Sprotte, the 22G Braun Spinocan and the 22G M. Schilling spinal needles were ***τ*** = 72 s; ***τ*** = 152 s and ***τ*** = 111 s, respectively.

**Fig. 4:**
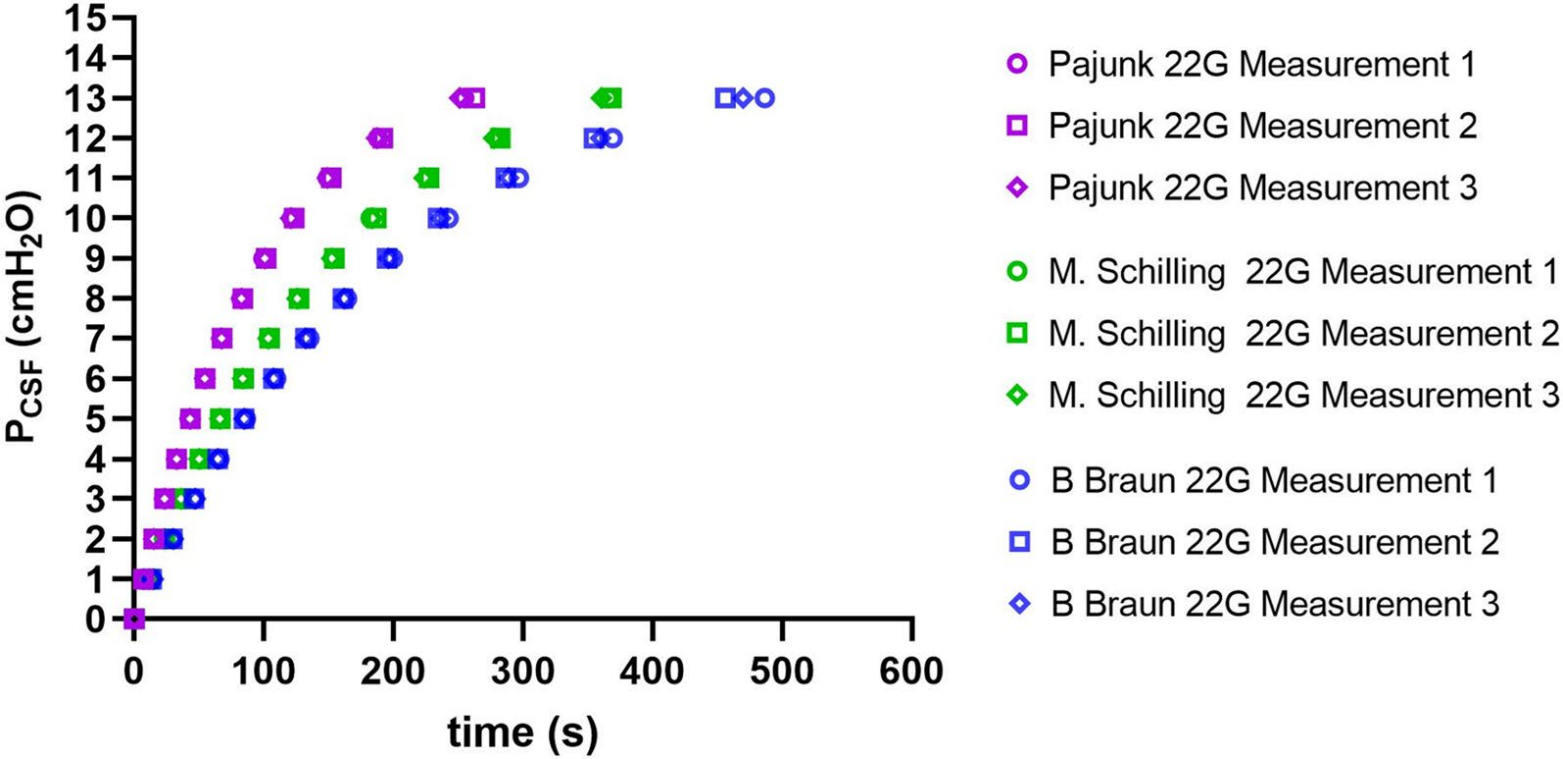
The behaviour of spinal needle-spinal manometer assembly is exponential. 22G Pajunk Sprotte, 22G B Braun Spinocan and 22G M. Schilling needles connected to a manometer with a final equilibrium of 13cmH2O is displayed. The graph is the plot of data from Table 2

First-order differential equation of the form given by the Eq. 6 was verified by non-linear regression fitting for each needle, To do this, spinal needles were connected with the manometer and P_CSF_ in the reservoir was set to 13cmH_2_O as shown in Fig. 2. The fluid rise in the manometer was monitored. The measurements are displayed in Table 2. For the same pressure level, it takes in average of 256 s for 22G Pajunk Sprotte needle to reach equilibrium and 364 s and 470 s for the 22G M. Schilling and 22G Braun Spinocan spinal needles, respectively. Given that 22G Pajunk needle has the lowest time constant (***τ*** = 72 s), as expected had the lowest equilibration time. Statistics for the data presented in Table 2 and illustrated in Fig. 4 is given in supplementary Table 1 (non-linear least square fitting).

**Table 2:** Spinal needles performances under 13 cmH_2_O pressure

Measurements	22G Pajunk Sprotte			22G M. Schilling			22G B Braun Spinocan		
	M1	M2	M3	M1	M2	M3	M1	M2	M3
P (cmH_2_O)	Time (s)	Time (s)	Time (s)	Time (s)	Time (s)	Time (s)	Time (s)	Time (s)	Time (s)
1	7477	7297	7664	11,223	11,484	11,529	13,843	13,414	14,705
2	15,262	15,119	15,226	23,453	23,559	23,418	29,669	29,356	30,154
3	23,532	23,608	23,465	36,503	36,263	36,098	46,654	46,242	47,304
4	32,746	32,903	32,833	50,713	50,764	50,099	65,669	64,521	64,483
5	42,85	43,497	43,014	66,54	66,495	66,246	86,268	84,756	84,804
6	54,429	54,859	54,507	84,133	84,582	83,735	109,424	107,42	107,443
7	67,493	67,913	67,464	103,875	104,351	103,339	135,146	132,06	132,327
8	82,794	83,742	82,705	126,741	127,224	125,766	164,203	161,072	161,821
9	99,915	101,705	100,722	153,524	154,463	152,368	199,339	195,063	196,323
10	121,72	123,534	120,855	182,494	186,791	183,667	242,336	234,635	236,528
11	149,557	152,16	149,105	225,71	227,57	223,957	296,737	286,846	288,835
12	188,186	191,823	187,137	280,662	282,595	277,612	369,026	354,984	359,79
13	254,348	262,977	251,124	365,752	368,164	360,552	486,537	456,29	469,991

Measurement results were curve fitted and compared with the theoretical ones to verify the exponential character of the time course of pressure rise from zero to 13 cmH_2_O in the manometer (Fig. 4).

The residual differences between predicted and true values were calculated for each data point, giving a RMS error of 0.68 cmH_2_O with the 22G Pajunk Sprotte; 0.43 cmH_2_O with the 22G B Braun Spinocan and 1.18 cmH_2_O with the 22G M. Schilling.

## Verification of the model using published data

To further verify that each needle/manometer combination had a unique time constant, data from other researchers were used. In the reference paper by Carson *et. al.*, constant CSF pressures of 12 cm CSF and 24 cm were generated using an artificial CSF solution, at 37°°C, with the assumption of µ_CSF_ = 1.10^–5^ (cm CSF s), to simulate a patient lying in the lateral position [2, 19]. A large reservoir of artificial CSF ensured that the pressure within the system remained constant throughout the tests. We calculated the flow rate *Q* for each needle by using the measurements from Carson et al.; i.e. by inserting the total mass *m*_*CSF*_ of fluid drops collected over a fixed period of 10 min [19]. Using these data (ref. [15] Table 2) we calculated the resistances to flow *R* by making use of Eq. 1 for Sprotte 24G Atraumatic, Spinocan 22G Quincke, BD 22G Quincke, BD 22G Whitacre and Sprotte 22G Atraumatic needles. Resistances to flow for these needles are shown in Table 3.

**Table 3:** Resistances to flow for the spinal needles and time constants calculated for P = 12 cmH_2_O (From data of the reference paper [15])

Needle type	R (cm s/ml)	*τ* (s)
Sprotte 24G Atraumatic	2413.41	142.9
Spinocan 22G Quincke	1421.05	66.7
BD 22G Quincke	1064.04	55.6
BD 22G Whitacre	907.56	38.5
Sprotte 22G Atraumatic	667.7	25

**Fig. 5:**
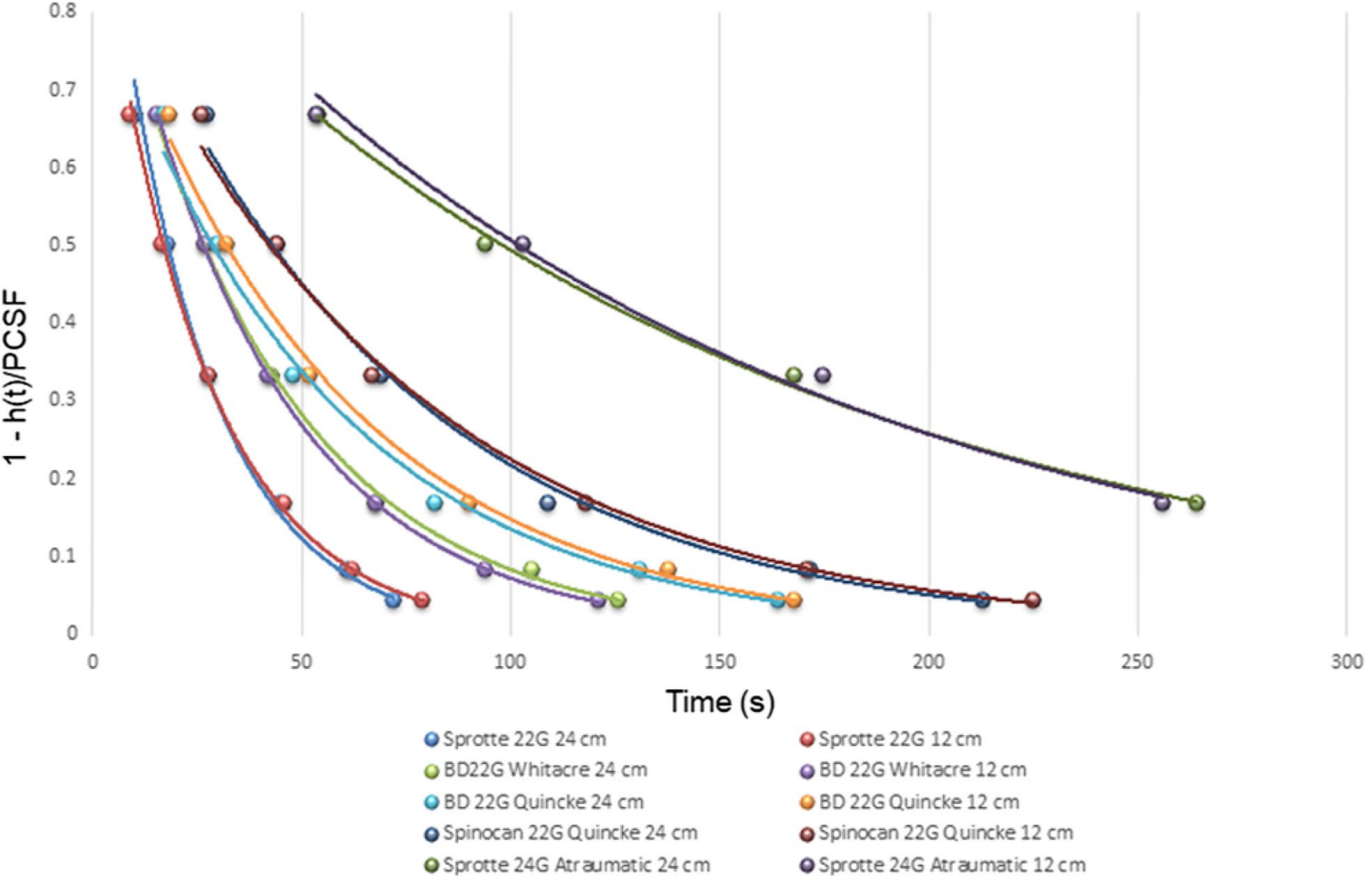
The time periods for the manometer to read specific percentages of the equilibrium pressures were similar, independent of the equilibrium pressure. From data of the reference paper [15].

Referring to the same paper (Table 3 in the reference paper), we reproduced times to 33,3%, 50%, 66,7%, 83,3%, 91,7% and 95,8% of the equilibrium pressure to display the time course of the CSF column rise in the manometer, for different types of needles (Fig. 5). We tested an exponential fitting for pressure rises in the manometer with each needle; a first-order differential equation of the form given by Eq. 6 was observed. As seen from Fig. 5, the time periods for the manometer to read specific percentages of the equilibrium pressures were similar, independent of the equilibrium pressure [15]. The regression coefficients R^2^ were equal or better than 0.99, in all cases.

For the verification of Eq. 5, we plotted the time constants *τ* against the resistances to flow* R*, with the assumption of *µ*_CSF_ = 1.10^–5^ (cm CSF s). The viscosity is unlikely to play a role in this case; even with high CSF protein and a pleocytosis. The viscosity of CSF and saline are similar and do not have a significant impact on CSF flow [15, 17]. The linear regression revealed the following relationship: τ = 0.067*R*-21.01, with the slope of 0.067 representing the cross-sectional area of a manometer of 3 mm diameter. Although the bore size of the manometer was not specified in the reference paper, a personal communication with Rocket® Medical revealed that internal diameter of their manometer was 3.05 mm ± 0.08 mm.

## Discussion

Indirect measurement of ICP via lumbar puncture with the use of a spinal manometer may not yield correct *P*_*CSF*_ results due to prolonged times required to obtain an accurate pressure value. Equilibrium pressure may be underestimated in circumstances where spinal manometry procedure is terminated prematurely, with the wrong assumption that equilibrium pressure is reached. *P*_*CSF*_ is an important parameter in clinical management and diagnosis of several neurological diseases including hydrocephalus, IIH and demyelinating disorders. These diseases may present with overlapping symptoms such as headache and visual defects [25–27]. Therefore, a quick and accurate assessment of *P*_*CSF*_ is essential. Our novel technique overcomes the caveats of spinal manometry allowing rapid and accurate estimation of *P*_*CSF*_.

In CSF pressure measurements via lumbar puncture, two parameters are of most importance: the needle flow rate of CSF at normal *P*_*CSF*_ to facilitate collection of at least 2 mL/min implying a small *R*; and the needle response to transduce the 90% of *P*_*CSF*_ accurately and quickly in less than 1 min. The length and the bore, hence the gauge of the needle, determine the resistance to flow. We have shown that time to respond to any CSF pressure level, can be computed by a first-order modelling of the measurement system, with the time constant calculated as the product of the needle *R* and the spinal manometer bore area.

The validity of the first-order model was tested 22G needles of different brands and data from the literature with a range of needle types from 24 to 22G. It is reported that spinal needles with a size of 20G and 22G can take from 1 to 5 min to reach equilibrium pressure and smaller needles such as 25G can take up to half an hour [2, 21]. Different equilibrium times were reported in the literature for spinal needles with identical outer diameter [15, 22]. This discrepancy was caused by the fact that needles of same gauge had different inner diameters. For the 22G needles used in our study had a range of τ from approximately 72 s to 152 s with a manometer of 3,7 mm diameter. For the 22G needles used by Carson et al., in combination with a manometer with a bore size of 3 mm, τ=25–66 seconds. For large *τ*, a lower P_CSF_ could be targeted to reduce measurement time, the improvement in measurement time would be in a ratio of approximately 7 to 1, when the measured pressure is reduced from 99% of P_CSF_ to 50% of P_CSF_. The viscosity is unlikely to play a role here; even with high CSF protein and a pleocytosis, viscosity of CSF and saline were not very different and do not impact appreciably on CSF flow [17]. The model has low-pass filter characteristics with time constants ranging from 25 s. to 152 s. corresponding to cut-off frequencies between 0.0011 Hz and 0.0063 Hz. The measurement system cannot respond to relatively faster CSF physiological rhythm changes caused by the (0.3 Hz) respiratory and (> 1 Hz) cardiovascular activities. However, such small changes bear no diagnostic value when measuring the CSF pressure.

The CSF measurement system of spinal needle and spinal manometer was compatible with the first-order model to predict the time required to reach the equilibrium CSF pressure in the manometer. A time constant τ was defined as the product of the resistance to flow of the needle with the bore area of the manometer divided by the dynamic viscosity of CSF: τ= *RA/ρ*_*CSF*_*.* Hence, each needle/manometer combination was expected to have a unique time constant. The time constant concept was tested on spinal needles with the same gauge (22G) from different brands. Spinal needles used in the published data was also considered for verification of the model. Curve fitting of the manometer readings were accomplished with regression coefficient R^2^ ≥ 0.99, when determining the measurement time constants. Measurement times required for a given percentage of *P*_*CSF*_ were independent of the final equilibrium level, in accordance with the experimental findings. CSF pressure values measured at reduced times could easily be interpolated to their equilibrium level; because the speed of measurement is very important for the maximum comfort of the patient. CSF pressure measurement times can be greatly minimized by selecting lower percentages of the equilibrium level by determining the time constant before the measurement. Therefore, we strongly recommend that spinal needle producers declare the resistance to flow *R* parameter in their data sheet. Similarly, spinal manometer manufacturers must always specify their bore diameters. This is a major limitation for our study since our spinal needle-spinal manometer assembly presented with a first-order differential equation requires precise inner diameter measurements of the manometer and the needle to provide accurate *P*_*CSF*_ values. It is not possible for a clinician to measure inner diameters of the needle and the manometer prior to performing an LP. Another limitation is that although this technique provides fast and accurate *P*_*CSF*_ measurement, the upward movement of the CSF might be too fast in certain spinal needle-spinal manometer combinations rendering the observation more difficult. Recent comparisons with electronic manometers and intravenous giving sets used in infusion therapy had shown that spinal column manometer is still the best alternative for pressure measurements during lumbar puncture [18, 22, 23]. Non-invasive ICP measurement has been of great interest to researchers for several decades however, none of these techniques are advanced enough to replace the invasive gold standard methods [4, 28]. One reason for this could be the low accuracy of the gold standard *P*_*CSF*_ measurement via LP which is used as a reference to compare non-invasive parameters.

## Conclusion

In this study, the spinal needle-spinal manometer combination was modeled with a first-order differential equation and a time constant was determined for each needle/manometer combination representing a unique constant as a predictor of the equilibrium pressure. Development and validation of our novel method was conducted in a simulated environment using a CSF model filled with an artificial fluid resembling CSF characteristics which served as an alternative to laboratory animals. We have found that measurement times required for a given percentage of *P*_*CSF*_ are independent of the final equilibrium level and waiting for CSF to reach the equilibrium height is not necessary. With this novel technique, it is possible to obtain *P*_*CSF*_ values with high accuracy within seconds. Given the importance of ICP in clinical management and diagnosis of neurological diseases, improvement of *P*_*CSF*_ measurement with our novel technique hold the potential to revolutionize the ICP estimation. As a future work, the model will be further tested for validation in the clinical settings.

## Supplementary Information

**Additional file 1: Table S1: **Non-linear least square fitting (n=42 for all needle types).

## Data Availability

The datasets used and/or analysed during the current study will be made available from the corresponding author on reasonable request.
