# Supplementary material for: A novel approach to CSF pressure measurement via lumbar puncture that shortens the measurement time with a high level of accuracy
Source: BMC Neurosci. 2023 Jun 18;24:34. doi: 10.1186/s12868-023-00805-4 (PMC10278321; doi:10.1186/s12868-023-00805-4)
Supplement: Supplementary file 1 — Additional file 1: Table S1: Non-linear least square fitting (n=42 for all needle types). [file 12868_2023_805_MOESM1_ESM.docx]

Additional file 1: Table S1: Non-linear least square fitting (n=42 for all needle types)

| **22G Pajunk Sprotte** |  |  |  |  |
| --- | --- | --- | --- | --- |
| Goodness of Fit | Measurement 1 | Measurement 2 | Measurement 3 | Global |
| Degrees of Freedom |  |  |  | 39 |
| R squared | 1 | 0.9999 | 0.9999 | 0.9999 |
| Sum of Squares | 0.00578 | 0.02272 | 0.01325 | 0.04175 |
| RMSE |  |  |  | 0.03191 |
| **22G M. Schilling** |  |  |  |  |
| Goodness of Fit | Measurement 1 | Measurement 2 | Measurement 3 | Global |
| Degrees of Freedom |  |  |  | 39 |
| R squared | 1 | 0.9999 | 1 | 1 |
| Sum of Squares | 0.005907 | 0.01248 | 0.01116 | 0.02955 |
| RMSE |  |  |  | 0.02685 |
| **22G B Braun Spinocan** |  |  |  |  |
| Goodness of Fit | Measurement 1 | Measurement 2 | Measurement 3 | Global |
| Degrees of Freedom |  |  |  | 39 |
| R squared | 0.9997 | 0.9998 | 1 | 0.9998 |
| Sum of Squares | 0.05854 | 0.03806 | 0.008695 | 0.1053 |
| RMSE |  |  |  | 0.05068 |
